# Number of cells in parathyroid tissue in primary hyperparathyroidism cases and its relationship with serum calcium value

**DOI:** 10.1097/MD.0000000000027530

**Published:** 2021-11-19

**Authors:** Mirkhalig Javadov, Emrah Karatay, Kivilcim Ulusan, Adnan Ozpek, Oğuz Idiz, Mete Duren, Serkan Sari, Firat Demircan, Gulderen Demirel, Husniye Dagdeviren, Ayse Yigit, Fahrettin Kelestimur, Erhan Aysan

**Affiliations:** a Yeditepe University, Faculty of Medicine, Department of General Surgery, Turkey; b Marmara University Pendik Training and Research Hospital, Department of Radiology, Turkey; c Health Sciences University Istanbul Hospital, Department of General Surgery, Turkey; d Health Sciences University, Umraniye Hospital, Department of General Surgery, Turkey; e Acibadem Maslak Hospital, Department of General Surgery, Turkey; f Health Sciences University, Başakşehir Cam ve Sakura Hospital, Department of General Surgery, Turkey; h Yeditepe University, Faculty of Medicine, Department of Internal Medicine, Turkey; g Yeditepe University, Faculty of Medicine, Department of Immunology, Turkey.

**Keywords:** number of cells, parathormone, phosphorus, primary hyperparathyroidism, serum calcium

## Abstract

**Background:**

The relationship between serum calcium (Ca) level to serum parathyroid hormone (PTH), phosphorus (P) levels and tissue properties of the parathyroid gland is unknown in primary hyperparathyroidism cases. Revealing this relationship may be useful for understanding the etiopathogenesis of primary hyperparathyroidism and determining the time of treatment.

**Methods:**

Ninety patients (71 females, 19 males, age range; 27–73 years, average age; 46) who underwent single gland excision with the diagnosis of primary hyperparathyroidism were studied. The patients were divided into 2 groups as serum Ca level <12 and serum Ca level ≥12. Age and sex of the patients, mean cell number of the gland, mean volume of the gland, serum levels of PTH, P, and histopathologic type of hyperplasia were evaluated.

**Results:**

The mean cell number per cubic centimeter is 22.9 (10–220 range) million in all glands. Serum Ca level was <12 in 82 (91.1%) of the patients, and ≥12 in 8 (8.9%) cases. Mean cell number of the gland, mean volume of the gland, existence of cystic hyperplasia of the gland, serum levels of PTH and P were statistically significant between the 2 groups (*P* < .001, *P* < .001, *P* < .05, *P* < .001, *P* < .05 respectively).

**Conclusion:**

In primary hyperparathyroidism cases serum Ca level is not related to age and sex but directly related to proportionals to the cell number and volume of the gland and serum levels of PTH, inversely related to cystic hyperplasia and serum levels of P. Early surgical intervention should be planned since the serum Ca level will be high in large adenomas with a noncystic radiological appearance.

## 1 Introduction

Hyperparathyroidism is a serious clinical condition that causes various resorptive bone diseases, atherosclerosis, renal calculosis, renal failure, and various destructive and functional gastrointestinal diseases.^[1]^ Ninety-five percent of hyperparathyroidism cases are primary hyperparathyroidism. In 90% to 95% of primary hyperparathyroidism cases, the disease is in 1 gland, while the other glands are healthy. From a histopathological point of view, almost all of the cases are adenomatous hyperplasia and the cancer frequency is less than 1%.^[1–3]^ As is well known, hyperplasia means an increase in the number of cells in a tissue. There is no information in the literature about the number of chief cells in primary hyperparathyroidism cases. The relationship between this number and demographic data such as the patient's age, gender, gland diameter, histopathological diagnosis of the gland as well as serum parathyroid hormone (PTH), calcium (Ca) and phosphorus (P) values were not revealed before. In this study, cell count was performed in the parathyroid tissue excised from patients with primary hyperparathyroidism, the relationship between chief cells number and the demographic data of the patient and the tissue were evaluated.

## 2 Materials and methods

### 2.1 Study design and data source

A prospective, multi-center clinical study was planned, and the approval of the local ethics committee was obtained. All patients were verbally informed about the study and their written consents with wet signature were obtained. Ninety patients (71 females, 19 males, age range; 27–73 years, mean age; 46) who underwent parathyroidectomy with the diagnosis of primary hyperparathyroidism between April 2018 and April 2020 in 4 different centers were included in the study. Inclusion criteria for the study; being over the age of 18, having normal serum vitamin-D level, serum Ca level being 10.6 mg/dL and above, having a single gland disease in the parathyroid, and not having a secondary or tertiary cause that may cause hyperparathyroidism. Serum Ca, PTH and phosphorous levels were measured in biochemistry laboratories of all 4 hospitals. Exclusion criteria; being under the age of 18, low serum vitamin D level, serum Ca level below 10.6 mg/dL even if serum PTH level is high, presence of disease in more than 1 gland in the parathyroid, a secondary or tertiary cause that may cause hyperparathyroidism. The relationship between age, gender, serum PTH, Ca, P values, mean cell number of whole gland, mean cell number per cubic centimeter, mean volume of the gland, and histopathological diagnosis of the gland were evaluated.

Before the ethical committee application we consulted a biostatistician for sample size calculation. He commented that a minimum of 65 cases was required based on power analysis using 0.05 accuracy and 0.95 power. Although the number of our cases exceeded 65, our study was planned as 2 years, so we continued to work until the end of this period in order to obtain more reliable results.

### 2.2 Study population and measurement

The patients were divided into 2 groups. Those with serum Ca between 10.6 and 12 mg/dL (high serum Ca group) and those with serum Ca level ≥12 mg/dL (very high serum Ca group). Standard open surgical technique was used with Kocher incision for all patients, and the surrounding tissue on the excised pathological parathyroid tissue were cleaned with careful dissection on the operating table. Then the tissue was divided into 2 equal parts. Half of the tissue was placed in formaldehyde suspension for histopathological examination and sent to the pathology laboratory. The other half of the tissue was placed in tubes containing sterile phosphate buffered saline. These tubes were placed in a thermostatic container containing dry ice and transferred to parathyroid research laboratory. The tissue was transferred to the cell culture cabinet there. A piece of 1 cm^3^ of tissue was excised under sterile conditions by measuring it with a ruler. This piece was cut into small pieces with a scalpel (mechanical crushing). It was then passed through a 10 μm diameter filter. It was centrifuged at 2500 rpm for 5 minutes. After the supernatant was discarded, the pellet was suspended in 1 mL phosphate buffered saline, and cell count was made by a flow cytometry device.

### 2.3 Statistical analysis

Descriptive parameters are presented as mean, standard deviation and percentages. Statistical analysis was performed with IBM SPSS 24 (Armonk, NY, USA), one-way ANOVA test was used for evaluation of the statistical significance between more than 2 subgroup analysis and a repeated ANOVA test was used for the specifically significant difference in time within the group. Tukey HSD and Bonferroni test were used for multiple comparisons. *P* < .05 was accepted as statistically significant.

## 3 Results

Serum Ca level was between 10.6 and 12 mg/dL in 82 (91.1%) of the patients, and serum Ca level was ≥12 mg/dL in 8 (8.9%). Mean age, female/male rate, mean cell number per cubic centimeter parameters were not statistically different between the 2 groups (*P* > .05). Mean cell number of the gland, mean volume of the gland, blood levels of PTH, Ca, and P were statistically significant between the 2 groups (*P* < .001, *P* < .001, *P* < .001, *P* < .001, *P* < .05, respectively, Table 1).

**Table 1:** Demographic and numeric results of the cases.

	All cases (n = 90)	Ca between 10.6 and 12 mg/dL cases (n = 82)	Ca ≥12 mg/dL cases (n = 8)	*P*
Mean age	46	47.5	44.5	>.05
Female/male rate	4.17	4.46	3	>.05
Mean cell number per cm^3^	22.9	22.3	23.2	>.05
Mean cell number of the gland	34.9 million (range 10–220)	32.7 million (range 10–69)	125.8 million (range 77–220)	<.001
Mean volume of the gland (cm^3^)	1.52 (range 0.5–6.4)	1.46 (range 0.5–4.4)	5.40 (range 2.7–6.4)	<.001
Mean serum PTH (pg/mL)	135.6 (range 102–255)	132.0 (range 102–180)	228.7 (range 210-255	<.001
Mean serum Ca (mg/dL)	11.2 (range 10.6–13.5)	11.0 (range 10.6–11.7)	12.5 (range 12–13.5)	<.001
Mean serum P (mg/dL)	2.7 (range 2.0–3.5)	2.7 (range 2.2–3.5)	2.1 (range 2–2.3)	<.05

Histopathological examination; Adenomatous hyperplasia without cystic areas was detected in 78 (86.6%) cases, and adenomatous hyperplasia with cystic areas in 12 (13.4%) cases. Malignancy or suspected malignancy was not detected in any of the cases. While the mean number of cells was 27.7 million in 78 cases without cystic areas and only with adenomatous hyperplasia, the average cell number in 12 cases with cystic hyperplasia was 20.1 million (*P* < .05). All cases with cystic hyperplasia were included in the group in which serum Ca level was between 10.6 and 12 mg/dL.

## 4 Discussion

An important parameter in assessing the functional capacity of an organ is its volume. The most concrete example of this is the liver. In patients who are planned to have liver resection for any reason, the maximum amount of liver volume that can be resected is calculated with volumetric examination.^[4,5]^ The volume of the small intestine remaining after small bowel resections is of vital importance. As the volume decreases, fluid electrolyte imbalance and the risk of mortality due to nutritional deficiency increase.^[6]^ It is known that lung-related infections are common in people with small spleen volume for various reasons (partial resection due to trauma, congenital hypoplasia, etc).^[7]^ The remaining gastric volume in patients who underwent sleeve gastrectomy due to obesity is related to the amount and speed of weight loss.^[8]^

As the size of the organ decreases, the sensitivity of volumetric evaluation decreases.^[9]^ In the evaluation of functional capacities of small-sized organs such as pituitary, ovary and parathyroid, cell number may be more sensitive data than volume. Knowing the total number of cells in an organ such as the pituitary gland may not provide useful data because the pituitary is composed of cell groups that secrete different hormones in different functional subunits (anterior pituitary and posterior pituitary).^[10]^ Another example are the ovaries. Since the ovaries act as a reservoir for the ovum, the cell number of the reservoir itself is not of primary importance. The number of ovums in the ovaries is determinant in evaluating the fertility capacity of a mammalian female and in the diagnosis of female type infertility.^[11]^

The parathyroid can be regarded as a model organ in which the number of cells can be valuable in functional evaluation because it is the smallest organ in the human body with an average diameter of 5 mm, consists of a single functional unit (chief cells) and secretes only 1 hormone (PTH). However, unfortunately, no studies have been conducted on this subject.

Congenital hypoparathyroidism is a very rare occurrence and constitutes approximately 5% of all hypoparathyroidism. In these individuals, congenital parathyroid glands are either absent or the number of cells are too low to provide normocalcemia.^[12]^ It is not known how many parathyroid cells are required to prevent this occurrence. On the other hand, it is not known how many total parathyroid cells are in a healthy person or how many parathyroid cells are in 1 parathyroid gland.

Knowing the parathyroid cell count has many clinical benefits. It is important that parathyroid tissues are not damaged and protected during thyroid surgery. There is an aphorism that leaving only 1 healthy parathyroid tissue behind in thyroid and parathyroid surgery is sufficient to prevent hypoparathyroidism, but there is no clinical research on this subject. As a matter of fact, it is impossible to conduct such a study due to ethical reasons.^[13–15]^

Parathyroid allotransplantation is an effective treatment method in the treatment of permanent hypoparathyroidism and has been used more frequently in recent years. However, for these transplants it is not known how many parathyroid cells should be transplanted to the patient. In some sources in the literature, 50 million cells are preferred, but this number is empirical and is not based on any clinical data.^[16,17]^

In the literature, there is no data on the number of cells in healthy parathyroid tissue, as well as on cell numbers in primary, secondary, and tertiary hyperparathyroidism cases. Our aim in this study was to reveal the cell number and related parameters in cases with primary hyperparathyroidism.

The main parameter determining the complications in cases of primary hyperparathyroidism is serum Ca level. Therefore, we divided our primary hyperparathyroidism patients into 2 groups as high serum Ca level (10.6–12 mg/dL) and very high serum Ca level (≥12 mg/dL). The elevation of serum Ca levels is not related to the age, gender and mean cell number per centimeter per gland of the patient. However, as the serum Ca level increases, the gland volume and blood levels of PTH increase, and the serum P value decreases.

### 4.1 Limitations

In radiological imaging, it can be predicted that as the diameter of the diseased parathyroid tissue increases, the serum Ca level and thus the risk of complications will increase. An exception is the presence of cystic hyperplasia in the gland. In this case, serum Ca level may be relatively low.

## 5 Conclusions

In cases with primary hyperparathyroidism, the mean cell number per cubic centimeter is 22.9 million, and this value is not related to age and sex. As the gland volume increases, the number of cells it contains increases and as a result, serum PTH and Ca increase and P decreases. The number of cells in the gland is directly proportional to noncystic adenomatous hyperplasia and inversely proportional to cystic adenomatous hyperplasia.

Early surgical intervention should be planned since the serum Ca level will be high in large adenomas. If ultrasonographic evaluation reveal noncystic radiological appearance serum Ca and/or end organ damage risk levels will be more serious.

## Author contributions

For all authors – study concept and design, acquisition of data, analysis and interpretation, drafting of manuscript, critical revision of the manuscript for important intellectual content and study supervision.

**Conceptualization:** Mirkhalig Javadov, Emrah Karatay, Oguz Idiz, Serkan Sari, Husniye Dagdeviren, Erhan Aysan.

**Data curation:** Mirkhalig Javadov, Emrah Karatay, Adnan Ozpek, Mete Duren, Firat Demircan, Gulderen Demirel, Ayse Yigit, Fahrettin Kelestimur, Erhan Aysan.

**Formal analysis:** Mirkhalig Javadov, Emrah Karatay, Kivilcim Ulusan, Oguz Idiz, Serkan Sari, Firat Demircan, Gulderen Demirel, Husniye Dagdeviren, Fahrettin Kelestimur, Erhan Aysan.

**Investigation:** Mirkhalig Javadov, Emrah Karatay, Kivilcim Ulusan, Adnan Ozpek, Oguz Idiz, Mete Duren, Firat Demircan, Ayse Yigit, Fahrettin Kelestimur, Erhan Aysan.

**Methodology:** Mirkhalig Javadov, Emrah Karatay, Kivilcim Ulusan, Adnan Ozpek, Oguz Idiz, Serkan Sari, Gulderen Demirel, Husniye Dagdeviren, Ayse Yigit, Fahrettin Kelestimur, Erhan Aysan.

**Project administration:** Mirkhalig Javadov, Adnan Ozpek, Mete Duren, Firat Demircan, Gulderen Demirel, Fahrettin Kelestimur.

**Resources:** Kivilcim Ulusan, Oguz Idiz, Serkan Sari, Husniye Dagdeviren.

**Software:** Mirkhalig Javadov, Emrah Karatay, Kivilcim Ulusan, Adnan Ozpek, Oguz Idiz, Mete Duren, Serkan Sari, Gulderen Demirel, Husniye Dagdeviren, Ayse Yigit, Erhan Aysan.

**Supervision:** Mirkhalig Javadov, Emrah Karatay, Kivilcim Ulusan, Adnan Ozpek, Mete Duren, Firat Demircan, Gulderen Demirel, Ayse Yigit, Erhan Aysan.

**Validation:** Mirkhalig Javadov, Emrah Karatay, Kivilcim Ulusan, Serkan Sari, Firat Demircan, Husniye Dagdeviren.

**Visualization:** Mirkhalig Javadov, Kivilcim Ulusan, Adnan Ozpek, Oguz Idiz, Mete Duren, Firat Demircan, Ayse Yigit, Fahrettin Kelestimur, Erhan Aysan.

**Writing – original draft:** Mirkhalig Javadov, Serkan Sari, Gulderen Demirel, Fahrettin Kelestimur, Erhan Aysan.

**Writing – review & editing:** Mirkhalig Javadov, Emrah Karatay, Mete Duren, Serkan Sari, Gulderen Demirel, Erhan Aysan.
